# Using multimodal ultrasound including full-time-series contrast-enhanced ultrasound cines for identifying the nature of thyroid nodules

**DOI:** 10.3389/fonc.2024.1340847

**Published:** 2024-08-29

**Authors:** Hanlu He, Junyan Zhu, Zhengdu Ye, Haiwei Bao, Jinduo Shou, Ying Liu, Fen Chen

**Affiliations:** ^1^ Department of Ultrasound, Ruijin Hospital, Shanghai Jiao Tong University School of Medicine, Shanghai, China; ^2^ Department of Ultrasound, The First Affiliated Hospital of Zhejiang Chinese Medical University, Hangzhou, China; ^3^ Department of Ultrasound, First Affiliated Hospital, School of Medicine, Zhejiang University, Hangzhou, China; ^4^ Department of Ultrasound, Sir Run Run Shaw Hospital, School of Medicine, Zhejiang University, Hangzhou, China

**Keywords:** thyroid nodules, ultrasonography, risk assessment, machine learning, radiomics

## Abstract

**Background:**

Based on the conventional ultrasound images of thyroid nodules, contrast-enhanced ultrasound (CEUS) videos were analyzed to investigate whether CEUS improves the classification accuracy of benign and malignant thyroid nodules using machine learning (ML) radiomics and compared with radiologists.

**Materials and methods:**

The B-mode ultrasound (B-US), real-time elastography (RTE), color doppler flow images (CDFI) and CEUS cines of patients from two centers were retrospectively gathered. Then, the region of interest (ROI) was delineated to extract the radiomics features. Seven ML algorithms combined with four kinds of radiomics data (B-US, B-US + CDFI + RTE, CEUS, and B-US + CDFI + RTE + CEUS) were applied to establish 28 models. The diagnostic performance of ML models was compared with interpretations from expert and nonexpert readers.

**Results:**

A total of 181 thyroid nodules from 181 patients of 64 men (mean age, 42 years +/- 12) and 117 women (mean age, 46 years +/- 12) were included. Adaptive boosting (AdaBoost) achieved the highest area under the receiver operating characteristic curve (AUC) of 0.89 in the test set among 28 models when combined with B-US + CDFI + RTE + CEUS data and an AUC of 0.72 and 0.66 when combined with B-US and B-US + CDFI + RTE data. The AUC achieved by senior and junior radiologists was 0.78 versus (vs.) 0.69 (*p* > 0.05), 0.79 vs. 0.64 (*p* < 0.05), and 0.88 vs. 0.69 (*p* < 0.05) combined with B-US, B-US+CDFI+RTE and B-US+CDFI+RTE+CEUS, respectively.

**Conclusion:**

With the addition of CEUS, the diagnostic performance was enhanced for all seven classifiers and senior radiologists based on conventional ultrasound images, while no enhancement was observed for junior radiologists. The diagnostic performance of ML models was similar to senior radiologists, but superior to those junior radiologists.

## 1 Introduction

In clinical practice, thyroid nodules are detected in up to 65% of the general population, of which approximately 90% are benign (1). The histological type of thyroid cancer that accounts for 84% is papillary thyroid cancer, which is the most common and least aggressive type (2). Global cancer statistics in 2020 (3) showed thyroid cancer is responsible for 586,000 cases in the world and ranks 9^th^ in incidence.

Ultrasound is the most important diagnostic image for thyroid nodules. Thyroid imaging report and data system (TI-RADS) criteria have been widely used clinically as risk stratification (4–8). TI-RADS only including B-mode ultrasound (B-US) information, whereas ultrasound technology offers multimodal imaging, including real-time elastography (RTE), color doppler flow images (CDFI), and contrast-enhanced ultrasound (CEUS). CEUS provides real-time dynamic observation of microvascular perfusion, which contributes to increased diagnostic accuracy (9, 10). The Bethesda System for Reporting Thyroid Cytopathology (TBSRTC) after fine-needle aspiration (FNA) applications is a well-established method for obtaining the diagnosis (11). The malignancy of thyroids nodules is evaluated by radiologists using multimodal ultrasound imaging. However, the conclusions of current studies on the additional diagnostic value of multimodal ultrasound are inconsistent and controversial (7, 12–14).

With its advancement, artificial intelligence (AI) has begun to reach or surpass human experts in medical imaging (15, 16) and has been applied to diagnose diabetic retinopathy, strokes, and breast lesions (17–19). Radiomics is a method to extract numerous quantitative parameters from standard-of-care medical imaging to obtain multidimensional information and mine high-throughput features that cannot be recognized by human eyes (20–22). It gains importance in thyroid research such as identifying benign and malignant thyroid nodules, predicting lymph node metastasis and disease-free survival of thyroid cancer (23–26). In addition, radiomics based on machine learning (ML) is reported to have also been applied to liver and breast medical fields (27–29). Recently, several studies have been conducted to evaluate the nature of thyroid nodules conducted based on ML (30–33). All of these studies used B-US images as input images, and some added shear-wave elastography (SWE), RTE, or CEUS images. It was found that a small amount of research analyzing the entire thyroid CEUS cines or the integrated information hidden behind ultrasound multimodal imaging (34).

Therefore, the conventional ultrasound images and CEUS cines of thyroid nodules were analyzed, and different image combinations were used to build ML models in the present study to explore the clinical value of thyroid multimodal ultrasound, especially CEUS. In the meanwhile, radiologists were invited to perform the discriminative readings of conventional ultrasound images and CEUS videos. Additionally, a comparison of diagnostic performance was made between radiologists and algorithms, and among different ML models, to find a more accurate method of improving clinical diagnosis.

## 2 Materials and methods

### 2.1 Study design and patients

Having been approved by the Institutional review board of the two participating centers, informed consent was waived for this retrospective study. The initial population consists of 71 patients with thyroid nodules who underwent CEUS at the First Affiliated Hospital of Zhejiang Chinese Medical University from September 2018 to January 2022, and 171 patients with thyroid nodules who underwent CEUS at the First Affiliated Hospital of Zhejiang University School of Medicine from December 2021 to January 2022.

Following were the criteria for inclusion: before starting treatment, patients with TI-RADS 4 or 5 category thyroid nodules should undergo the following procedures: (1) a CEUS examination;(2) a fine-needle aspiration biopsy; and (3) measurement of the maximal diameter of a thyroid nodule, which was between 0.4 and 1.5 cm. The following were the exclusion criteria: (1) patients with incomplete clinical or imaging data; (2) CEUS cines were of poor quality; and (3) nodules with Bethesda categories I, III, IV, and V.

**Figure 1:**
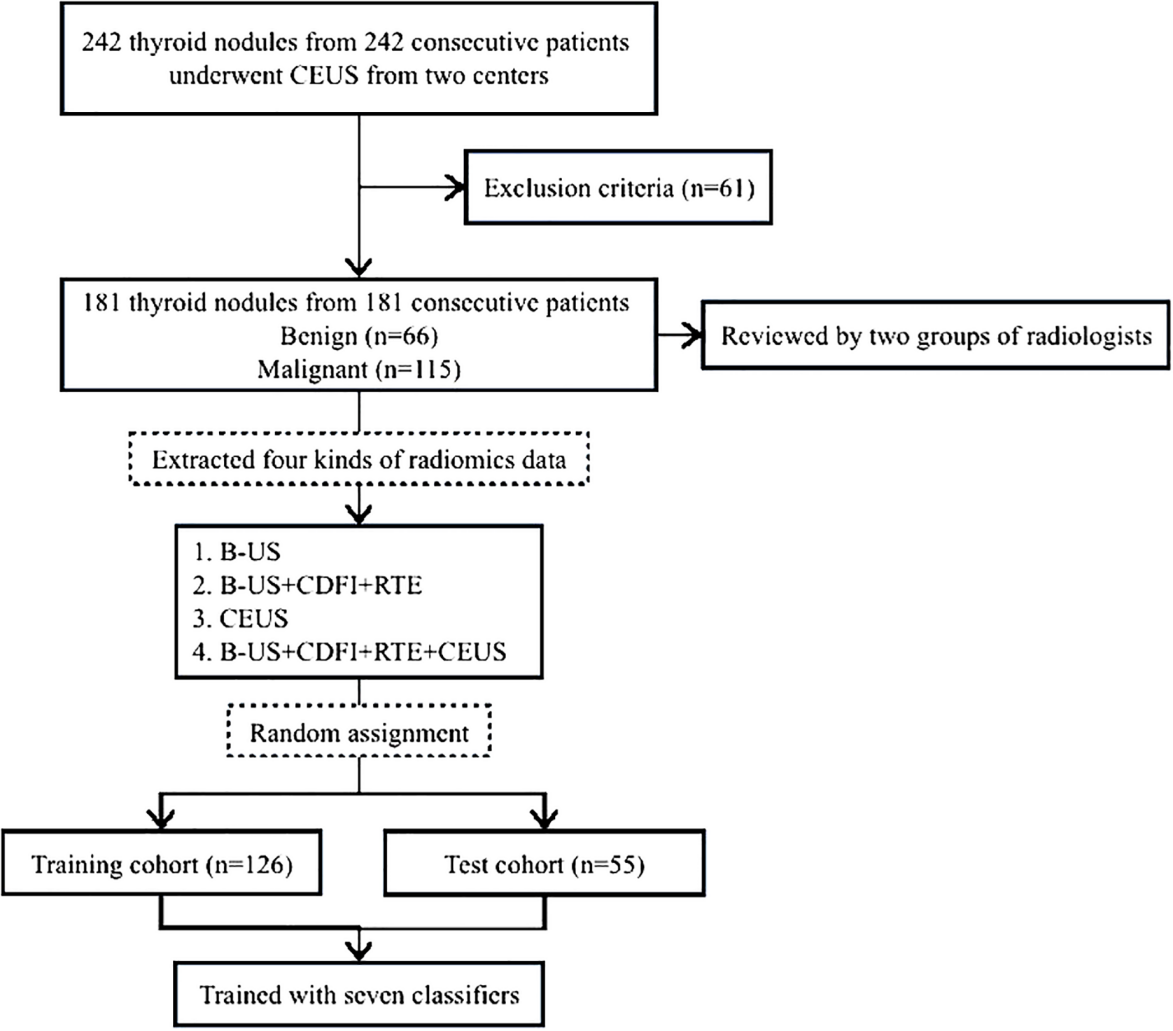
Flowchart of patient selection for the study. B-US, B-mode ultrasound; RTE, real-time elastography; CDFI, color doppler flow images; CEUS, contrast-enhanced ultrasound.

181 patients with thyroid nodules, including 66 benign nodules and 115 malignant nodules, were included in the final thyroid dataset. They were randomly divided into a training cohort of 126 patients and a testing cohort of 55 patients at a 7: 3 ratios. **Figure 1** displays a thorough flowchart of the patient selection process for this study.

### 2.2 Ultrasound image acquisition

In this study, CEUS was performed using two ultrasound instruments: Esaote Mylab 90 and Philips EPIQ 7. The imaging parameters were adjusted by board-certified and experienced radiologists who performed the CEUS examination and acquired the cines. For each examination, the image settings, including the time-gain compensation, the focal position, the dynamic range, the output power, the mechanical index and so on, were optimized.

After getting images of thyroid nodules in B-US, CDFI and RTE modes, the second-generation ultrasound contrast agent (SonoVue, Bracco SpA) was used during the CEUS which was made of sulfur hexafluoride gas microbubbles. It contained 1.5 mL of contrast agent for thyroid CEUS. Following the injection of the contrast agent diluted with normal saline into the antecubital vein within 1 s, 5–10 mL of 0.9% normal saline was subsequently flushed. The target lesion on the largest plane was continuously observed and captured for 60 seconds from the start of injection, and the entire CEUS imaging process was documented on an ultrasound workstation with the Digital Imaging and Communications in Medicine (DICOM) format.

All study-related videos were completed and recorded by two radiologists (C.F. And Y.ZD.), both of whom have over 15 years of experience in evaluating thyroid CEUS.

### 2.3 ROI delineation

**Figure 2:**
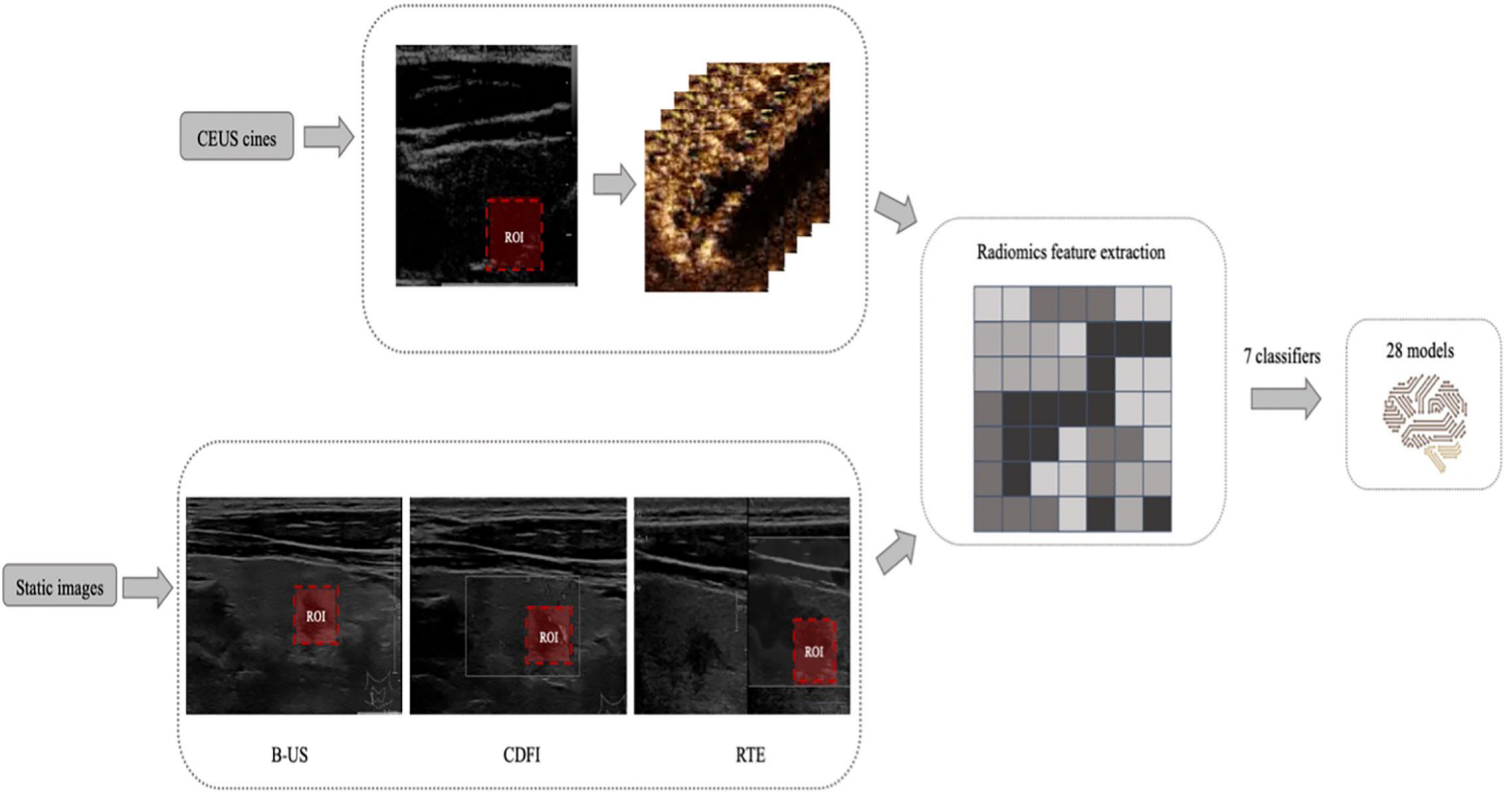
Example of delineating regions of interest (ROIs) on a contrast-enhanced ultrasound (CEUS) frame and 3 static images in B-mode ultrasound (B-US), real-time elastography (RTE), and color doppler flow images (CDFI) modes. And the design process of all machine learning (ML) models.

All thyroid CEUS videos from two hospitals were converted into AVI format, while static B-US, CDFI, and RTE images were converted into JPGE format. The radiologists first reviewed the complete video to observe the lesion boundaries. Then, the rectangular region of interest (ROI) was delineated on a CEUS frame and 3 static images in B-US, CDFI and RTE modes separately using Labelme (version 3.21.1), including the entire lesion and part of the surrounding tissues, and the images were stored in JSON format. The bounding box remained unchanged on every frame of the CEUS cine (**Figure 2**).

All ROIs were manually delineated by a young radiologist (H.HL.) and then reviewed by another senior radiologist (C.F.), all of whom were blinded to the clinical and pathological data of the patients. Any disagreement among the radiologists was resolved by discussion until a consensus was reached.

### 2.4 Radiomics feature extraction and model building

Features were extracted from the ROI using PyRadiomics (version 3.0.1). Extracted texture features were calculated on the two-dimensional shape (9 features), first-order statistics (18 features), gray-level cooccurrence matrix (23 features), gray-level run-length matrix (16 features), gray-level size-zone matrix (16 features), neighboring gray tone difference matrix (5 features) and gray level dependence matrix (14 Features). A detailed definition of all image features can be found online. (http://pyradiomics.readthedocs.io/en/latest/features.html).

The minimum analysis time of a CEUS video is 1 minute; a rate of 18 frames per second was applied for a total of 1080 images. The difference between each picture is small and changes over time. Therefore, 1080 lines of radiomics features were extracted from the 1-minute video of each patient’s CEUS cine.

We applied 7 supervised ML algorithms; these classifiers were k nearest neighbors (KNN), random forest (RF), logistic regression (LR), Support Vector Machines (SVM), eXtreme Gradient Boosting (XGBoost), Gradient Boosting (GB) and Adaptive Boosting (AdaBoost). Four classes of radiomics data were used as the inputs of each ML model: B-US, B-US + CDFI + RTE, CEUS and B-US + CDFI + RTE + CEUS. Seven classification methods combined with four kinds of radiomics data to establish 28 (7× 4 = 28) models. Each of the 28 models was trained and 10-fold cross validated in the training set with scikit-learn. The receiver operating characteristic (ROC) curve and area under the ROC curve (AUC) were employed to evaluate the predictive accuracy of the radiomics signatures developed. The model that had the highest AUC value in the test dataset was selected as the final model.

### 2.5 Subjective evaluation

A total of four radiologists (S. JD., B.HW., L.Y., and Z.JY.) retrospectively reviewed static images and CEUS cines of patients with thyroid nodules. All radiologists were blinded to the clinical and pathological information of the patients and split into two groups: experienced radiologists (S. JD. and B.HW., with more than 15 years of clinical experience) and junior radiologists (L.Y. and Z.JY., with less than 10 years of clinical experience). None of the radiologists were involved in the CEUS examinations.

**Figure 3:**
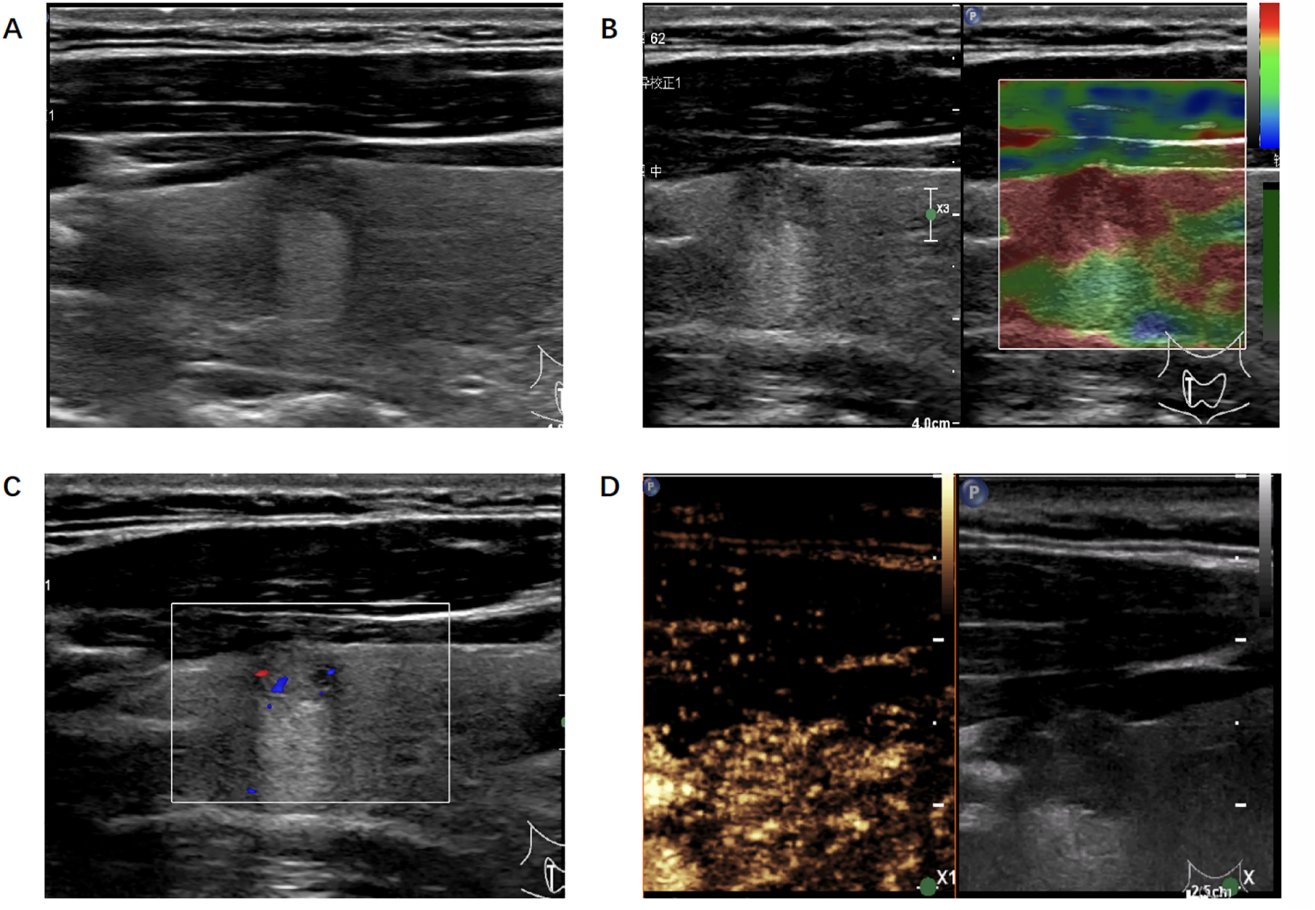
Ultrasonic image of the thyroid nodule which was misjudged by the radiologists but correctly predicted by the algorithm. **(A)** B-mode ultrasound showed a longitudinal section of the nodule. **(B)** Real-time elastography image of the nodule. **(C)** Color doppler flow image of the nodule. **(D)** The 30-second image of contrast-enhanced ultrasound in thyroid nodule.

Conventional ultrasound (including B-US, CDFI, and RTE) static images and CEUS cine clips were successively reviewed by two groups of radiologists (**Figure 3**). The radiologists assessed the possibility of malignancy of each lesion and diagnosed it as malignant or benign independently, based on the Thyroid Imaging Reporting and Data System (TI-RADS). In cases in which discrepancies existed within the group, a consensus was reached after discussion.

### 2.6 Statistical analysis

Student’s t test or the Mann-Whitney test, as appropriate, was used to compare continuous variables. The χ2 test was used to compare categorical variables. The AUCs were used to evaluate the probability of correct classification of benign and malignant nodules. Differences between AUCs were calculated using the DeLong test.

To evaluate the predictive performance of different models, sensitivity (SEN), specificity (SPE), accuracy (ACC), positive predictive value (PPV), negative predictive value (NPV) and F1 score were investigated. Data analysis was performed using SPSS (version 26.0) and PyRadiomics (version 3.0.1). All statistical tests were two-sided. Differences were considered significant at *p* < 0.05.

## 3 Results

### 3.1 Baseline characteristics

Baseline clinical and pathological data came from patients’ medical records, including age, sex, and lesion size (**Table 1**). A total of 181 patients were included in this study. Among the thyroid nodules, 66 (36.5%) nodules were benign, and 115 (63.5%) nodules were malignant. Among 117 female patients, benign nodules were found in 47 patients (40.2%) and malignant nodules in 70 patients (59.8%). Among 64 male patients, benign nodules were found in 19 patients (29.7%) and malignant nodules in 45 patients (70.3%). Patients with malignant thyroid nodules were younger than those with benign thyroid nodules(33.0-47.0y vs. 46.0-59.3y, *p* < 0.05). No difference was observed between benign and malignant thyroid nodules in size on B-US (0.57-0.89cm vs. 0.53-0.93cm, *p*>0.05).

**Table 1:** Characteristics of patients and images.

Characteristics	Benign	Malignant	*P*
Patients number	66 (36.5%)	115 (63.5%)	
Sex			
Female Male	47(40.2%) 19(29.7%)	70(59.8%) 45(70.3%)	
Age (years)			
Median	51.5	40.0	<0.05
Interquartile	46.0-59.3	33.0-47.0	
Size of lesions(cm)			
Median	0.68	0.66	>0.05
Interquartile	0.57-0.89	0.53-0.93	

### 3.2 Performance evaluation of ML models

**Figure 4:**
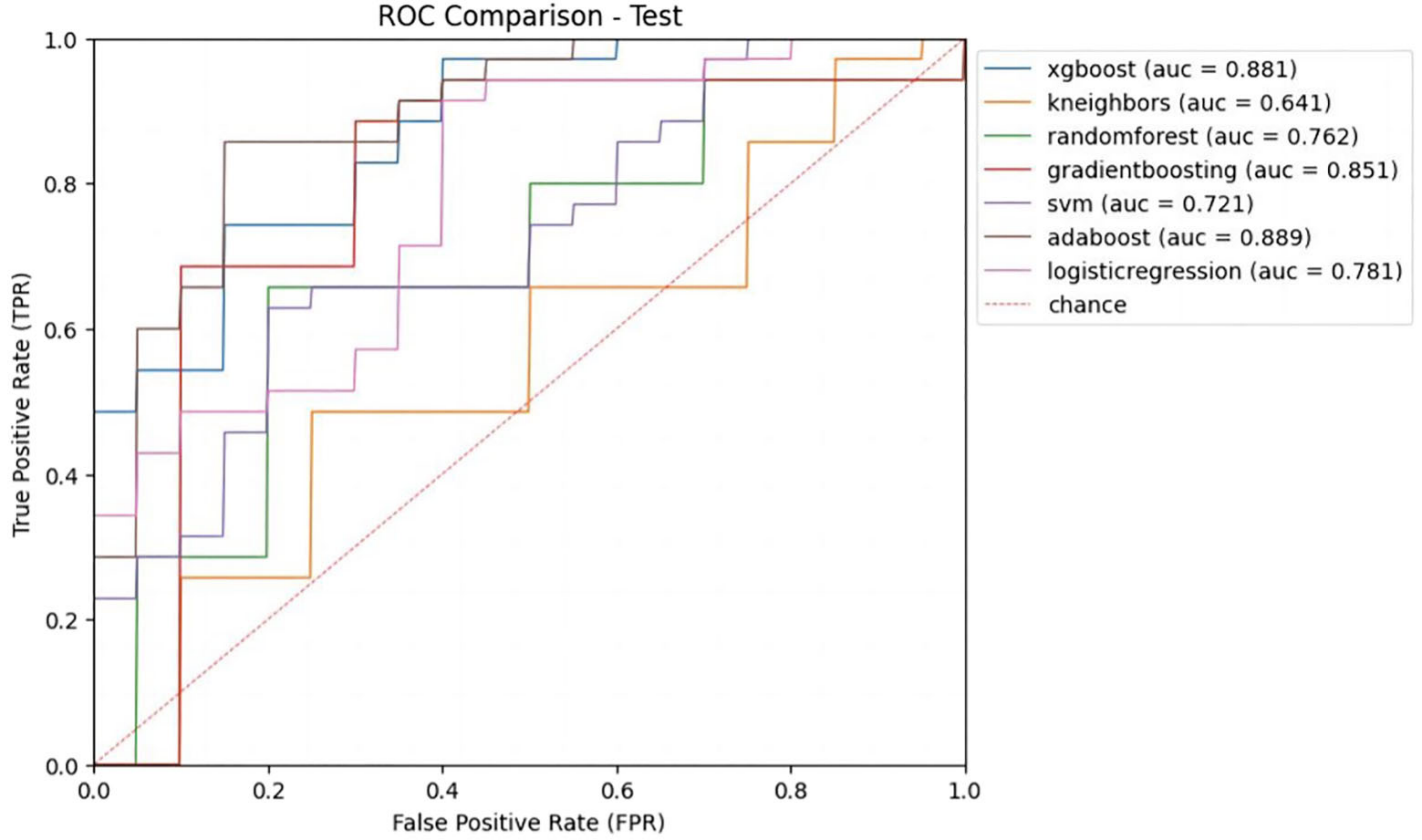
Receiver operating characteristic (ROC) curves of different ML models with B-US+CDFI+RTE+CEUS data combination in test cohort.

Inputting different radiomics features extracted from 4 types of image sets resulted in 28 models constructed with 7 different ML methods, and the AUCs of these models were evaluated in the test cohort (**Table 2**). The AUCs of B-US data combined with all 7 classifiers ranged from 0.55 to 0.72 and the AUCs of B-US + CDFI + RTE data combined with all 7 classifiers ranged from 0.58 to 0.71. According to this, we found that CDFI and RTE ultrasonic image data have a low classification value. For the CEUS data combined with all 7 classifiers, the AUCs of the models ranged from 0.65 to 0.83. Given the B-US+CDFI+RTE+CEUS data combined with all 7 classifiers, the AUCs of the models ranged from 0.64 to 0.89 (**Figure 4**).

**Table 2:** Comparison of the area under the receiver operating characteristic curves (AUCs) of different machine learning (ML) methods with different data combinations in test cohort.

Models	B-US	B-US+CDFI+RTE	*p*1 value	CEUS	B-US+CDFI+RTE+CEUS	*p*2 value
XGBoost	0.68	0.64	0.65	0.76	0.88	<0.05
SVM	0.55	0.67	0.14	0.83	0.72	0.49
RF	0.55	0.64	0.41	0.69	0.76	0.17
LR	0.63	0.64	0.84	0.81	0.78	0.12
KNN	0.61	0.71	0.19	0.65	0.64	0.39
GB	0.67	0.58	0.31	0.74	0.85	<0.05
AdaBoost	0.72	0.66	0.30	0.80	0.89	<0.05

XGBoost, eXtreme Gradient Boosting; SVM, Support Vector Machines; RF, random forest; LR, logistic regression; KNN, k nearest neighbors; GB, Gradient Boosting; AdaBoost, Adaptive Boosting. B-US, B-mode ultrasound; CDFI, color doppler flow images; RTE, real-time elastography; CEUS, contrast-enhanced ultrasound.

*p1*: B-US+CDFI+RTE compared with B-US.

*p2*: B-US+CDFI+RTE+CEUS compared with B-US+CDFI+RTE.

Among the 7 classifiers, only 4 (SVM, RF, LR, and KNN) achieved higher AUCs with B-US+CDFI+RTE data combined than with only B-US data alone (0.67 vs. 0.55, 0.64 vs. 0.55, 0.64 vs. 0.63, 0.71 vs. 0.61). There were 6 of 7 classifiers (XGBoost, SVM, RF, LR, GvB, and AdaBoost) that achieved higher AUCs with B-US+CDFI+RTE+CEUS data combined than with B-US+CDFI+RTE data combined (0.88 vs. 0.65, *p*<0.05; 0.72 vs. 0.67, *p >*0.05; 0.76 vs. 0.64, *p*>0.05; 0.78 vs. 0.64, *p >*0.05; 0.85 vs. 0.58, *p*<0.05; 0.89 vs. 0.66, *p*<0.05).

We obtained the predictive performance of 7 classifiers with B-US+CDFI+RTE+CEUS data in the test cohort (**Table 3**). Among them, XGBoost and AdaBoost achieved better performance. Their AUCs were 0.88 and 0.89, respectively, and both ACC was 0.82.

**Table 3:** Comparison of the predictive performance of different ML models with B-US+CDFI+RTE+CEUS data combination in test cohort.

Models	ACC	SEN	SPE	PPV	NPV	F1
XGBoost	0.82	0.94	0.60	0.81	0.86	0.87
SVM	0.60	0.66	0.50	0.70	0.46	0.68
RF	0.71	0.66	0.80	0.85	0.57	0.74
LR	0.75	0.83	0.60	0.78	0.67	0.81
KNN	0.64	0.86	0.25	0.67	0.50	0.75
GB	0.80	0.89	0.65	0.82	0.77	0.85
AdaBoost	0.82	0.94	0.60	0.81	0.86	0.87

XGBoost, eXtreme Gradient Boosting; SVM, Support Vector Machines; RF, random forest; LR, logistic regression; KNN, k nearest neighbors; GB, Gradient Boosting; AdaBoost, Adaptive Boosting. ACC, accuracy; SEN, sensitivity; SPE, specificity; PPV, positive predictive value; NPV, negative predictive value; F1, F1 score.

### 3.3 Performance evaluation comparison of algorithm and radiologists

**Figure 5:**
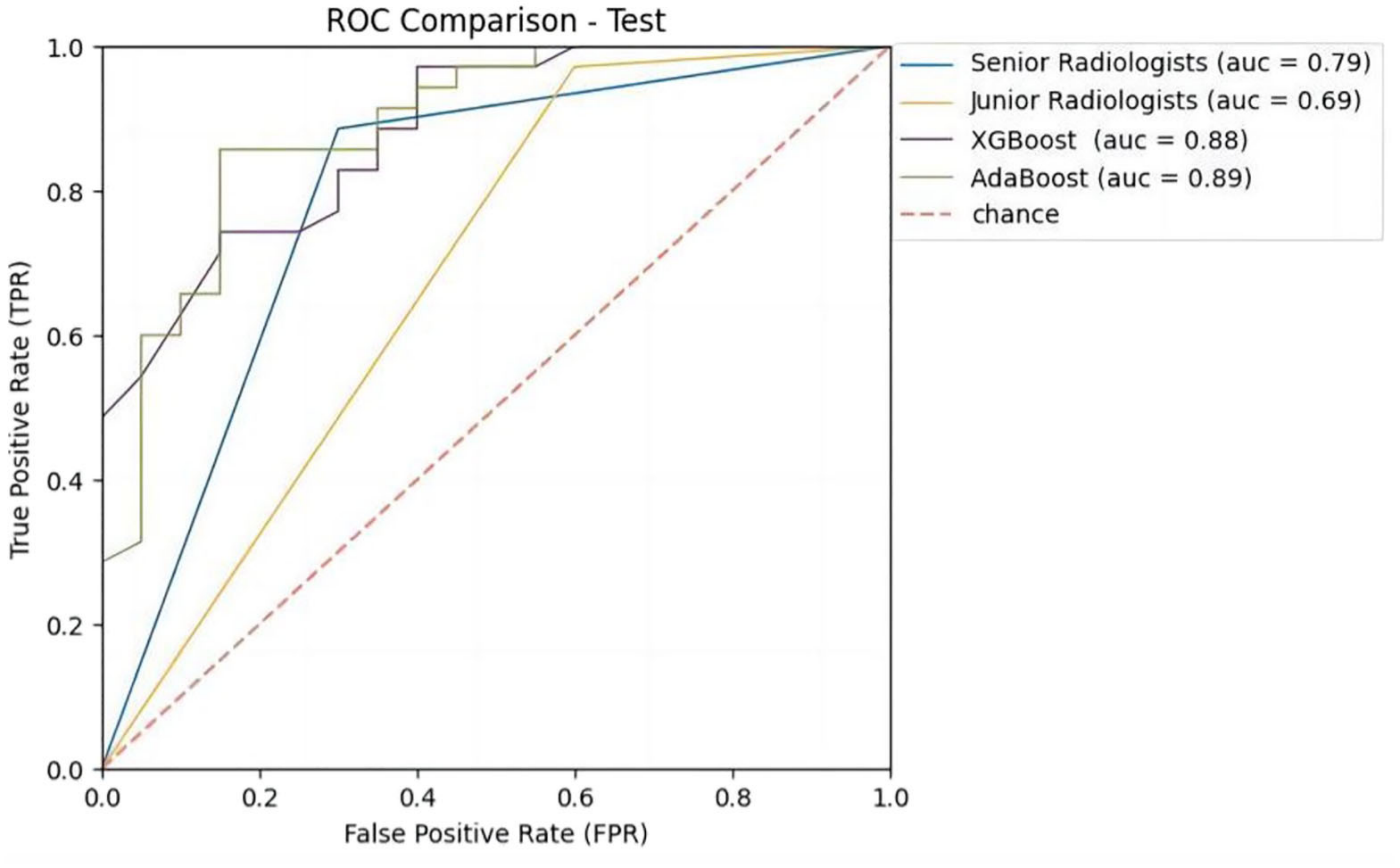
Receiver operating characteristic (ROC) curves of ML models and radiologists with B-US+CDFI+RTE+CEUS data combination in test cohort.

In order to compare the diagnostic performance with radiologists, we selected 2 classifiers (XGBoost and AdaBoost) with higher AUCs from the B-US+CDFI+RTE+CEUS data combination (**Table 4** and **Figure 5**). For both B-US and B-US+CDFI+RTE data combination, the AUCs of the 2 classifiers approximated those of junior radiologists but fell below those of senior radiologists. For the combination of B-US+CDFI+RTE+CEUS data, the AUCs of 2 classifiers exceeded those of junior and senior radiologists. In all 3 different kinds of combinations, the AUCs of senior radiologists were higher than the AUCs of junior radiologists (0.78 vs. 0.69, *p*>0.05; 0.79 vs. 0.64, *p*<0.05; 0.88 vs. 0.69, *p*<0.05).

**Table 4:** Comparison of AUCs of ML models and radiologists with different data combinations in test cohort.

	B-US	B-US+CDFI+RTE	*p*1 value	B-US+CDFI+RTE+CEUS	*p*2 value
XGBoost AdaBoost Senior Radiologists	0.68 0.72 0.78	0.64 0.66 0.79	0.65 0.30 0.32	0.88 0.89 0.88	<0.05 <0.05 0.16
Junior Radiologists	0.69	0.64	0.15	0.69	0.15
*p*3 value	0.45	0.15	/	0.86	/
*p*4 value	0.74	0.71	/	<0.05	/
*p*5value	0.18	<0.05	/	<0.05	/

XGBoost, eXtreme Gradient Boosting; AdaBoost, Adaptive Boosting; B-US, B-mode ultrasound; CDFI, color doppler flow images; RTE, real-time elastography; CEUS, contrast-enhanced ultrasound.

*p1*: B-US+CDFI+RTE compared with B-US.

*p2*: B-US+CDFI+RTE+CEUS compared with B-US+CDFI+RTE.

*p3*: AdaBoost compared with Senior Radiologists.

*p4*: AdaBoost compared with Junior Radiologists.

*p5*: Senior Radiologists compared with Junior Radiologists.

After adding the CDFI and RTE data, the AUCs of both classifiers and junior radiologists decreased compared to when only B-US data were available (0.64 vs. 0.68, *p*>0.05; 0.66 vs. 0.72, *p*>0.05; 0.64 vs. 0.69, *p*>0.05). When B-US+CDFI+RTE+CEUS data were combined, both classifiers and radiologists obtained higher AUCs than when B-US+CDFI+RTE data were combined (0.88 vs. 0.64, *p*<0.05; 0.89 vs. 0.66, *p*<0.05; 0.88 vs. 0.79, *p*>0.05; 0.69 vs. 0.64, *p*>0.05).

### 3.4 Performance evaluation comparison of algorithm and radiologists in difficult cases

Cases with disagreement after the discussion of senior radiologists or agreement but inconsistent with pathology results were defined as difficult cases. From 24 difficult cases in the test set, overall, the AUCs of the classifiers were closer to those of senior radiologists, and the AUCs of the classifiers were higher than those of junior radiologists.

With the addition of CDFI and RTE data, the AUCs of both classifiers and senior radiologists increased compared to use B-US data only (0.66 vs. 0.50, *p*>0.05; 0.56 vs. 0.55, *p*>0.05; 0.58 vs. 0.52, *p*>0.05), and then with the addition of CEUS data, the AUCs of both classifiers and senior radiologists increased also (0.80 vs. 0.66, *p*>0.05; 0.83 vs. 0.56, *p*<0.05; 0.66 vs. 0.58, *p*>0.05) (**Table 5**). In contrast, after adding reading CDFI, RTE, and CEUS imaging, the AUC of junior radiologists decreased instead.

**Table 5:** Comparison of AUCs of ML models and radiologists with different data combinations in 24 difficult cases of test cohort.

	B-US	B-US+CDFI+RTE	*p*1 value	B-US+CDFI+RTE+CEUS	*p*2 value
XGBoost AdaBoost Senior Radiologists	0.50 0.55 0.52	0.66 0.56 0.58	0.29 0.96 0.32	0.80 0.83 0.66	0.35 <0.05 0.41
Junior Radiologists	0.61	0.54	0.14	0.54	1.00
*p*3 value	0.85	0.89	/	0.17	/
*p*4 value	0.72	0.95	/	<0.05	/
*p*5 value	0.45	0.78	/	0.30	/

XGBoost, eXtreme Gradient Boosting; AdaBoost, Adaptive Boosting; B-US, B-mode ultrasound; CDFI, color doppler flow images; RTE, real-time elastography; CEUS, contrast-enhanced ultrasound.

*p1*: B-US+CDFI+RTE compared with B-US.

*p2*: B-US+CDFI+RTE+CEUS compared with B-US+CDFI+RTE.

*p3*: AdaBoost compared with Senior Radiologists.

*p4*: AdaBoost compared with Junior Radiologists.

*p5*: Senior Radiologists compared with Junior Radiologists.

## 4 Discussion

In our study, we finally built 28 ML-based radiomics models and the AUC of the best model was 0.89 when inputting multimodality ultrasound imaging. In the same situation, the AUC of senior radiologists was 0.88 and 0.69 for junior radiologists. We showed a case where the radiologist was wrong but the algorithm predicted correctly (**Figure 3**). The diagnostic performance of the algorithm and radiologists for identifying benign and malignant thyroid nodules differed. What’s more, the addition of CEUS data improved their diagnostic performance, implying that thyroid CEUS cines carry substantial information that can be mined and analyzed.

Cancer statistics for Chinese from 2016 indicated that thyroid cancer has grown considerably, ranking fourth among newly common cancers in women (35). The incidence of thyroid cancer has continued to increase in many countries, but its mortality rate has remained stable over the same period, suggesting that much of the increase is due to over-diagnosis, which accounts for 60-90% of detected cases of thyroid cancer in some countries (36, 37). Multiple methods are available for determining the nature of nodules, including multimodal ultrasonography, fine needle aspiration, and AI based non-invasive methods. Examinations that combine B-US, CDFI, RTE, and CEUS are preferred by radiologists for the assessment of thyroid nodules that appear to be malignant. B-US serves as the basis for the classification of malignancy of thyroid nodules. TI-RADS is commonly used in clinical work (38), enhancing diagnostic accuracy and reducing unnecessary biopsies (5, 39). The value of the following ultrasound techniques is in continuous exploration, with a number of studies highlighting and utilizing CEUS. As we know, CDFI detects blood flow inside and around thyroid nodules, while RTE indicates thyroid nodule hardness, increasing diagnostic accuracy (40). Several scholars have done studies on the correlation between ultrasound strain elastography (SE) and the size of thyroid nodules (41–44). Based on three diagnostic tools including SE, it was investigated that a cut-off value of 10 mm and 15 mm in the diameter of thyroid nodules may not be able to predict the degree of malignancy. Our study improves the possibility of recognizing the nature of thyroid nodules with diameters less than 15 mm by using ML combined with multimodal ultrasound images. CEUS can detect differences in blood distribution, as well as differences in hemodynamics between the tumor and the surrounding tissue (10).

Applying AI to evaluate and analyze image data is an emerging field (15–19, 24, 25). Based on our limited knowledge, we have not yet seen a study making full use of extensive information extracted from full-time sequences of thyroid CEUS cines. A study added CEUS to B-US images, featuring a single frame of the CEUS cine with peak enhancement intensity to represent the entire CEUS cine (30). Applying DL methods to the breast had already been done by our team (45). Now we explored whether ML methods would be more effective considering the data volume of thyroid nodules.

The DL-based method works like a “black box” and cannot clearly show the intermediate process. Meanwhile, this study’s sample data is small and is not suitable for deep learning methods such as neural networks, which require large amounts of data. Thus, ML-based methods were tried in this study. Seven methods in ML classification algorithms were selected. First, LR the most commonly used double classification methods in traditional statistical methods, can be used as a reference for the classification effect of this study. KNN was selected because it is a frequently used classifier and is easy to understand, which utilizes a training sample and predicts the new sample by majority voting on the results of the k-nearest points to the new sample. SVM and RF classifier are the most effective classification methods in ML. RF is an improvement on the bagging method, which reduces variance by building and averaging many trees to obtain an approximately unbiased model. SVM in the context of binary classification can be formulated as a model for finding a decision boundary that maximizes the margin between two data categories. GB, XGBoost, and AdaBoost belong to the Boosting family, which can promote weak learners to strong learners. AdaBoost emphasizes adaptability and constantly adds weak classifiers to boost by constantly modifying sample weights (increasing the weight of wrong samples and decreasing the weight of split pair samples). In addition, it simulates the clinical ultrasound decision-making path and analyze the four kinds of radiomics data in order with the above ML methods.

The results showed that AdaBoost classification, which combines several weak classifiers to create a superior classifier, was had the best effect. It outperformed the effects of experienced doctors. This may be because AdaBoost can enhance the accuracy of weak classifiers, is relatively robust to overfitting (under certain conditions), and its efficiency and adaptability make it a strong choice for classification tasks on small datasets. In the meanwhile, we found that the multimodal ultrasound model integrated with the shortest one-minute full-time-series CEUS cines yielded a maximum AUC of 0.89 and a maximum accuracy of 0.82. Such a model is more clinically relevant and can facilitate the diagnosis and treatment of thyroid nodules. Several factors make our study different from others. To distinguish between benign and malignant thyroid nodules, several thyroid radiomics research extracted multi-dimensional features from thyroid B-US images by SVM methods. The accuracy of the SVM models in these studies ranged from 75.9% to 98.3% (33, 46, 47). Park VY et al. extracted features based on thyroid B-US images and developed a linear prediction model, the best model yielded an AUC of 0.75 in the test set (48). While our best model with B-US images yielded an AUC of 0.72. When it comes to thyroid multimodal images, a study established ML‐assisted visual approaches and radiomics approaches based on B-US and SWE images to predict the malignancy of thyroid nodules (31). What’s more, Zhang B et al. demonstrated that both ML models and radiologists can lead to more reliable differentiation of benign and malignant nodules based on B-US combined with RTE images (32). However, in our study, when adding CDFI and RTE images, the predictive performance of both 3 classifiers (XGBoost, GB, AdaBoost) and junior radiologists seems to decline compared with B-US images only. Overfitting existed due to the weakened generalization ability of the model after the addition of CDFI and RTE feature samples. Meanwhile, the static images might have been less informative than the dynamic cines, while the junior radiologists did not interpret CDFI and RTE accurately enough. On the other hand, adding CEUS data to B-US, CDFI, and RTE data combination increased the AUCs of XGBoost, AdaBoost, GB, and junior radiologists. Compared to real clinical work, where radiologists routinely observe the features of thyroid nodules dynamically, static CDFI and RTE images have failed to accurately convey comprehensive information about the nodules. Simultaneously, CEUS cines deliver a larger amount of data and information than CDFI and RTE images.

After the inclusion of CEUS, the trend of AUC changed differently among ML models, senior radiologists, and junior radiologists. Junior radiologists were inexperienced in CEUS interpretation. On the contrary, senior radiologists were more experienced in CEUS interpretation owing to the long CEUS learning curve, which contains a lot of detailed domain knowledge. Comparatively, the ML methods captured the information accurately without the above-mentioned differences. It was shown in our results that the diagnostic performance of both over half of the classifiers and senior radiologists improved after adding CDFI and RTE data to the B-US data, while almost all classifiers and radiologists performed better after adding CEUS data.

In general, with each addition of multimodal data, especially CEUS data, the AUCs of models gradually increased. A conclusion could be drawn that B-US, CDFI, and RTE images are valuable, and the inclusion of CEUS cines can provide a higher level of value. In the discrimination of benign and malignant thyroid nodules, CEUS was of great help to both the algorithm and radiologists by improving their diagnostic performance. Out of 28 different classifiers, the classifier with better diagnostic performance surpassed the performance of junior radiologists but was similar to that of senior radiologists. This finding was similar to outcomes from other studies (49, 50). In 24 difficult cases, with the inclusion of CEUS, the ML models excelled senior radiologists and significantly outperformed junior radiologists, demonstrating the powerful ability to identify thyroid nodules when CEUS is integrated with the ML algorithm.

The current study still contained some limitations. First of all, only a small sample dataset was obtained for this study, and no additional medical research centers were combined to capture a more comprehensive sample. In addition, this study did not provide a separate external validation cohort; instead, internal validation was used. Third, the B-US, CDFI, and RTE images reviewed in this study were static and left features from multi-sections of thyroid nodules unconsidered. In follow-up studies, multimodal ultrasound dynamic video modeling and classification of thyroid nodules would have high clinical application value.

## 5 Conclusion

Multimodal ultrasound images including CEUS combined with the ML algorithm provide a better classification of thyroid nodules as benign or malignant, and CEUS optimizes the diagnostic performance of both algorithms and radiologists.

## Data Availability

The original contributions presented in the study are included in the article/supplementary material. Further inquiries can be directed to the corresponding author.
